# Pancreatic Cystic Neoplasms and Pregnancy: A Systematic Review of Surgical Cases and a Case Report of a Fully Laparoscopic Distal Pancreatectomy

**DOI:** 10.1097/SLE.0000000000001023

**Published:** 2021-12-09

**Authors:** Alessandro Fogliati, Mattia Garancini, Fabio Uggeri, Marco Braga, Luca Gianotti

**Affiliations:** * School of Medicine and Surgery, Milano-Bicocca University, Milan; † Department of Surgery, San Gerardo University Hospital, Monza, Italy

**Keywords:** pancreatic cyst, pregnancy, mucinous cystic neoplasm, laparoscopic distal pancreatectomy, solid pseudopapillary tumor

## Abstract

Mucinous cystic neoplasms and solid pseudopapillary neoplasms are the most common pancreatic tumors occurring in women of fertile age and in pregnant women. The aim of this study is to provide an updated literature review on this association and to present a fully laparoscopic resection of a pregnancy-associated pancreatic cystic neoplasm.

**Materials and Methods:**

A systematic literature review was performed using PubMed (MEDLINE), Scopus, Ovid, ISI Web of Science, and Google Scholar for searching. The syntax was (pancr*) AND (cyst*) AND (pregn*) AND (tumor). Only English-language articles describing pancreatic surgical resections were included.

**Results:**

Forty-seven case reports were included. The mean age of the patients was 29.6±5.3. Nine patients (20%) required emergency surgery, 4 (9%) due to cyst rupture, and 5 (11%) due to hemorrhage. Four patients (9%) suffered a miscarriage, and 2 (5%) opted for pregnancy termination; the rest of the women delivered a healthy newborn (86%, n=36). Thirty percent (n=14) of the resected neoplasms were malignant, and among mucinous cystic lesions, this raised to 45% (n=11). All patients diagnosed during the third trimester were resected postpartum, whereas 26/34 (76%) of patients diagnosed during the first 2 trimesters underwent surgery before delivery.

**Conclusions:**

The most worrisome complications in pregnancy-associated pancreatic cysts are bleeding or rupture. Mucinous cystic neoplasm has a tendency to grow during pregnancy. A postpartum resection was generally preferred when the cystic neoplasm was diagnosed during the third trimester. This report is the first to describe a fully laparoscopic pancreatic resection.

## Abstract 2

Supplemental Digital Content is available in the text.

In the last decades, accumulating evidence indicates that the extent utilization of advanced radiologic technologies correlates with an increased incidental reporting of the pancreatic cystic lesions.^1^ Cystic neoplasms of the pancreas include a wide range of lesions with benign, borderline, and malignant behavior. Among these neoplasms, mucinous cystic neoplasms (MCNs) and solid pseudopapillary neoplasms (SPNs) are commonly diagnosed in fertile age women. SPNs have the potential for local invasion, but after radical surgical resection, recurrence is rare.^2^,^3^ Mucinous cystadenomas are usually found in the tail of the pancreas and can present at a benign stage or after degeneration into cystadenocarcinomas.^4^,^5^ The ovarian-stroma component and the expression of estrogen and progesterone receptors is a plausible explanation for the association of sex and age and appearance of SPN and MCN, and the pregnancy-related hormonal changes may potentially account for faster growth.

The surgical treatment of an incidentally diagnosed pancreatic cystic neoplasm during pregnancy is challenging because it may jeopardize the health a young patient and her fetus. Given the disorder complexity, a multidisciplinary discussion should be undertaken, but the timing and the type of surgical approach often depends on the experience of a surgeon because of the lack of robust recommendations and guidelines on the topic.

A consistent number of case reports and case series^6^–^49^ described the treatment of pregnancy-associated pancreatic cystic (PAPC) lesions.

The aim of the present study is to describe a case of laparoscopic resection of a large MCN in a young woman after natural delivery and to provide an updated literature review focusing on the management and outcome of patients bearing pancreatic cystic lesions surgically treated during and after pregnancy.

## CASE REPORT

**FIGURE 1:**
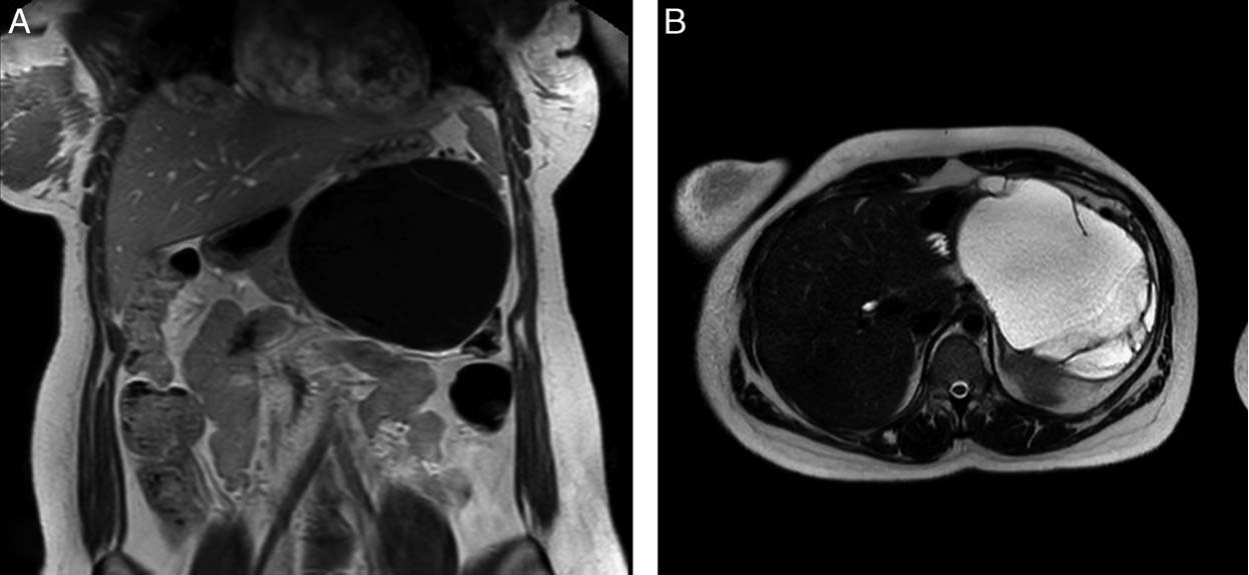
The 18×16 cm mucinous cystadenoma at the preoperative contrast-enhanced magnetic resonance imaging. A, Coronal section in T1 weighted. B, Transverse section in T2 weighted.

**FIGURE 2:**
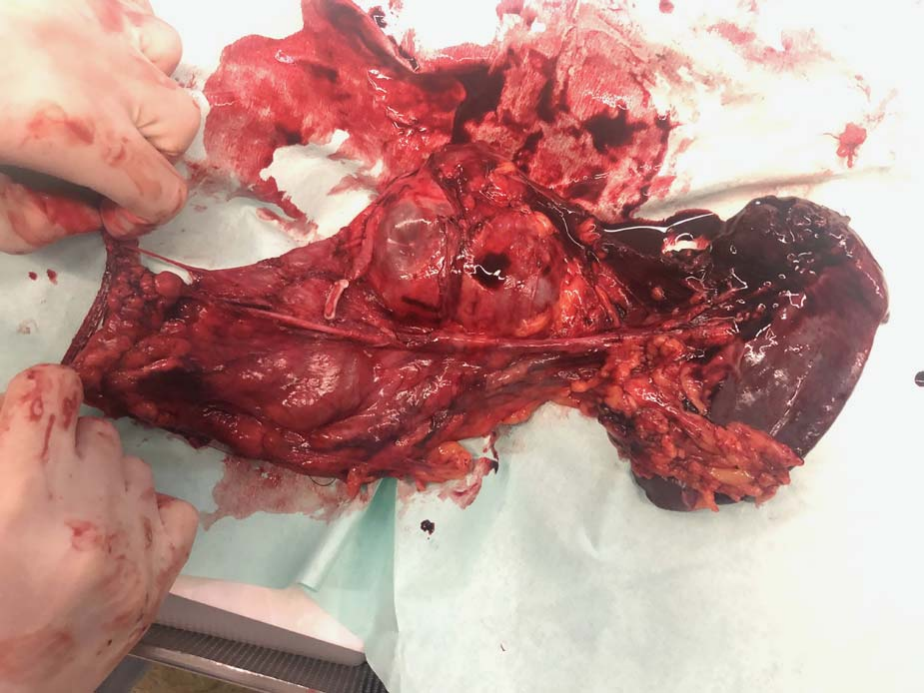
The mucinous cystadenoma after aspiration and extraction. On the left side, the resection margin on the pancreatic body is visible, and on the right side, the lesion extends toward the hilum of the spleen.

In October 2020, a 25-year old African woman in the 33rd gestational week presented at our institution for her first abdominal ultrasound. During the examination, an 18×16 cm pancreatic cystic lesion at the tail of the pancreas was described. The case was discussed in a multidisciplinary setting, and because of the proximity to the expected delivery date and the asymptomatic clinical scenario, it was decided to postpone any further diagnostic workup and surgical treatment. Four weeks after vaginal delivery of a healthy newborn, the patient underwent magnetic resonance imaging (Fig. 1) and endoscopic ultrasound with the aspiration of the cystic fluid for assay of carcinoembryonic antigen (CEA) and carbohydrate antigen 19-9 (CA 19-9) (9222 mcg/L, 589 U/mL, respectively). The radiologic features, the fluid analysis, the sex and age of the patient supported the diagnosis of an MCN. Despite the large volume of the cyst, the patient underwent a fully laparoscopic distal pancreatectomy with spleen removal using a 3-layer endoscopic staple closure of the pancreatic stump. The surgical specimen was then enveloped in a laparoscopic endo-bag, and the extraction was carried through a 5 cm supraumbilical laparotomy after the placement of a wound protector (Alexis; Applied Medical, Rancho Santa Margarita, CA). The enveloped specimen was surfaced through the laparotomy, the endo-bag opened, the fluid content aspirated without spillage, and the specimen eventually removed. Figure 2 shows surgical specimen after cyst aspiration and extraction. The key phases of the operation are shown in the enclosed video.

The postoperative course was uneventful, with the exception of the development of grade A (or biochemical leak) pancreatic fistula. The patient was discharged on postoperative day 15, and the pathology report confirmed the diagnosis of MCN with a low-grade dysplasia.

## MATERIALS AND METHODS

The present review of the literature was conducted using the Preferred Reporting Items for Systematic Reviews and Meta-Analyses guidelines.^50^

### Criteria for Inclusion in the Literature Review

Types of studies: Case series, case reports, and systematic reviews.

Types of participants: Female patients diagnosed with a pancreatic cystic neoplasm during pregnancy.

Types of interventions: Open or laparoscopic pancreatic resection or biopsy.

Types of outcome measures: Surgery and pregnancy-related morbidity.

### Search Methods for Identification of Studies

Electronic databases PubMed (MEDLINE), Scopus, Ovid, ISI Web of Science, and Google Scholar, were searched from January 1980 to February 2021. The search strategy used the following syntax: (pancr*) AND (cyst*) AND (pregn*) AND (tumor) AND ([literature AND review]. No geographic limits were applied, and only articles in English were considered. The reference list for each literature review was examined for missing studies within the scope of this review.

### Study Selection

Following the removal of duplicate references, 2 reviewers (A.F., M.G.) independently assessed the eligibility of each of the remaining preselected references by screening the titles, keywords, and abstracts according to the systematic review eligibility criteria. The reviewers independently assessed the full texts of all potentially relevant references for their eligibility. Following selection, discrepancies between the reviewers were resolved by discussion and, when a consensus could not be reached, a final decision was taken by the majority after consultation with a senior reviewer (L.G.). Reference lists of full-text articles were hand-searched to identify relevant publications that were missed. Inclusion criteria for study selection were publications reporting diagnosis and resection of pancreatic cystic lesions during pregnancy or within 90 days after delivery. The exclusion criteria were publications not reporting surgical outcomes.

### Data Extraction and Management

Data extraction was performed by a standard electronic spreadsheet with the reviewers independently extracting the data from all eligible articles. Extractions were compared and disagreements were resolved by discussion or by consultation with a third reviewer. The following data and information were extracted: year of publication, number of patients included, age at diagnosis, gestational age at diagnosis, associated symptomatology, tumor localization, diameter of the cyst, growing pattern during pregnancy, rupture of the cyst, tumor bleeding, type of surgical resection, timing of the surgical resection (before vs. after delivery), and outcome measurements (surgical and gestational morbidity).

## RESULTS

### Literature Selection

**FIGURE 3:**
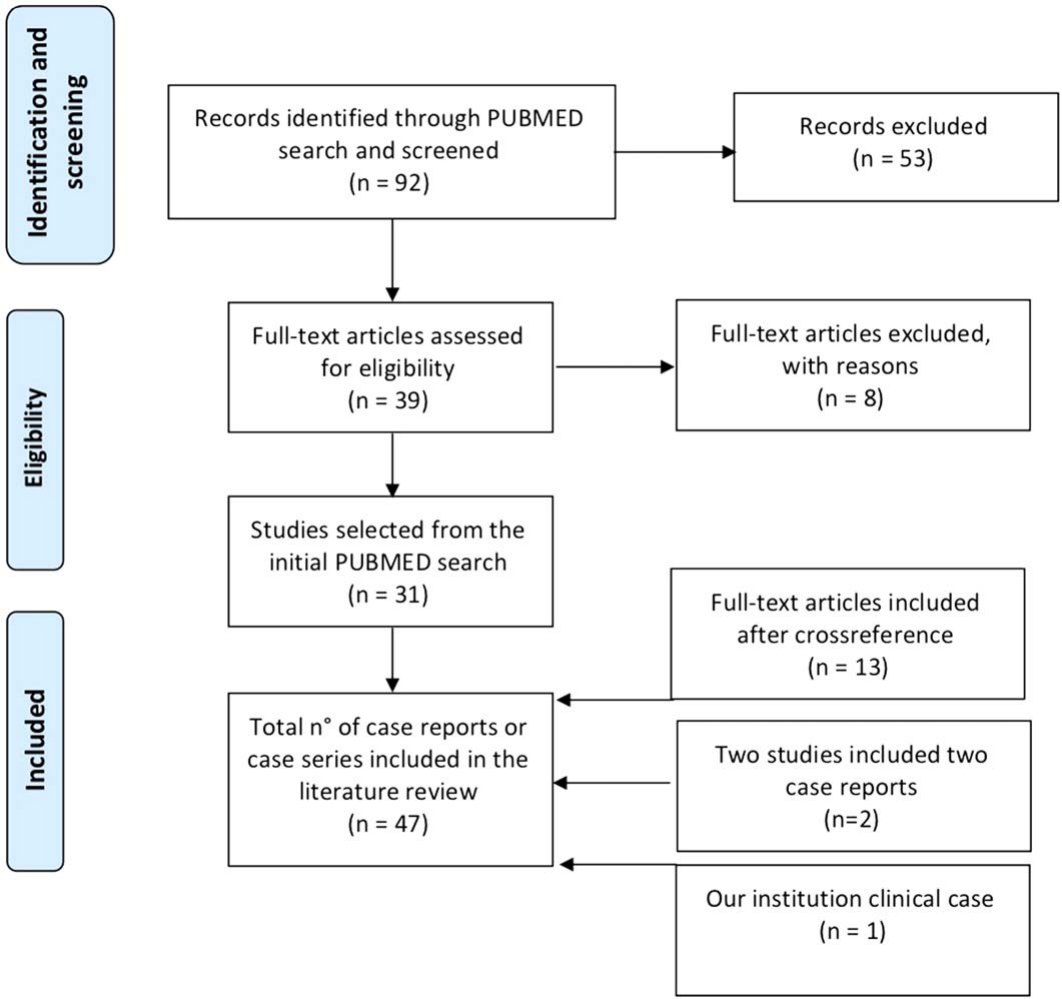
Flowchart for study selection.

The literature search identified 92 publications, 39 were selected after abstract reading, 8 case reports were excluded because not in English language or not pertinent to the review, and 31 of were included in this review. After cross-reference other 13 articles were identified and added to the study. Two of the included studies presented 2 clinical cases. Moreover, we included the present case. Overall, 47 cases were included in the present review. Detailed characteristics of the included publications are reported in Supplementary Table 1 (Supplemental Digital Content 1, http://links.lww.com/SLE/A302). The literature selection flowchart is depicted in Figure 3.

### Population Characteristics

The mean±SD age of women presenting with pancreatic cystic neoplasm was 29.6±5.3 and 53% of the patients were primigravidae. The mean gestational age was 16.9±9.3 weeks. Forty percent (n=18) of the patients were asymptomatic at the time of diagnosis. The most commonly reported symptoms were abdominal pain (47%; n=21), nausea (13%; n=6), and back pain (7%; n=3), only 1 patient (2%) presented with asthenia due to anemia^27^ and another (2%) presented at the emergency room in premature labor.^24^

The most common emergencies were cyst rupture and hemorrhage. Four cysts ruptured during pregnancy (9%), and 5 patients (11%) presented with hemorrhage, in 1 case, the bleeding was caused by concomitant splenic artery rupture without aneurismatic degeneration.^21^

Serum levels of tumor markers were underreported in the reviewed clinical cases. CA 19-9 was found elevated in 28% of the cases (n=5), while CEA was elevated in 15% of the cases (n=2). Six patients (14%) underwent endoscopic ultrasonography in their diagnostic workup. The majority of the cystic neoplasms were located in the body-tail of the pancreatic gland (78%; n=34). The mean maximum diameter of the pancreatic cysts was 136.3±41.5 mm, and 52% (n=22) of them increased in size during the pregnancy. Details on population and cyst characteristics are reported in Table 1.

**TABLE 1:** Population and Cyst Characteristics

	n (%)	Clinical Cases Reporting Data
Age (mean±SD) (y)	29.6±5.3	45
Primigravida	16 (53)	30
Gestational age at diagnosis (mean±SD) (wk)	16.9±9.3	40
Symptomatology		45
Asymptomatic	18 (40)	
Abdominal pain	21 (47)	
Nausea	6 (13)	
Back pain	3 (7)	
Asthenia	1 (2)	
Premature labor	1 (2)	
Endoscopic ultrasound	6 (14)	44
Elevated serum CA 19.9	5 (28)	18
Elevated serum CEA	2 (15)	13
Cyst location		44
Head	10 (23)	
Body/tail	34 (77)	

CA 19-9 indicates carbohydrate antigen 19-9; CEA, carcinoembryonic antigen.

### Surgical and Pregnancy Outcomes

The majority of patients were treated with a distal pancreatectomy (64%; n=27), 19% (n=8) received a pancreatoduodenectomy, in 7% (n=3) of the cases, a total pancreatectomy was performed, 7% (n=3) of patients received an enucleation, and 1 patient (2%) received an explorative laparoscopy due to the metastatic stage of the disease at presentation.^23^ Most of the patients were surgically treated during pregnancy, whereas 43% (n=20) received surgery after delivery. Only 2 surgical procedures were carried out with a laparoscopic approach.^23^ One was a laparoscopic peritoneal exploration, and the present one with a fully laparoscopic left resection. Nine patients (19%) required an emergency surgical procedure, 4 of them due to cyst rupture and 5 due to hemorrhage, while the remaining 38 (81%) patients received an elective procedure. Thirty-six (86%) pregnancies resulted in the delivery of a healthy newborn, 9 of them through vaginal delivery (21%), 12 through cesarean section (29%), and in 15 cases (36%), the delivery was through an unspecified route but with a healthy newborn. There were 4 (9%) cases of involuntary abortion and in 2 cases (5%) of voluntary abortion. Pathology reports showed a benign disease in 70% of the cases (n=33): 16 MCN (34%) with low-grade dysplasia, 2 MCN (4%) with high-grade dysplasia, 13 SPN (28%), 1 pancreatic hemangioma (2%), and 1 pancreatic lymphangioma (2%). Malignancy was reported in 30% (n=14) of the patients: 11 mucinous cystadenocarcinomas (23%), 2 invasive SPN (4%), and 1 invasive gastrointestinal stromal tumor (2%). MCN and SPN were the most frequent pancreatic neoplasms diagnosed during pregnancy, representing 61% and 32% of the cases, respectively.

All 4 ruptured cysts^7^,^24^,^33^,^48^ were MCN, 3 (75%) of them were malignant at the final pathology report. The mean diameter of the ruptured cysts was 140±45 mm. Cyst rupture always required emergency surgical treatment; in 2 (50%) patients, it was associated with delivery through a cesarean section. Among the 5 cystic tumors complicated by hemorrhage^10^,^21^,^27^,^30^,^47^ 2 were benign SPN (40%), 1 was a benign MCN (20%), 1 an invasive MCN (20%), and 1 a lymphangioma (20%). The mean diameter of the hemorrhagic cystic was 128.3±49 mm. All hemorrhagic cysts required emergency surgery resulting in 2 abortions (40%) and in a successful delivery in 3 cases (60%). Regarding postoperative pancreatic fistulae, only 1 biochemical leak and 1 grade B fistula were reported. Other postoperative complications reported were a case of spleen infarction after spleen preserving distal pancreatectomy and a case of death due to pulmonary embolism. A detailed description of the outcomes is reported in Table 2.

**TABLE 2:** Surgical Details, Pregnancy Outcomes, and Pathology Reports

	n (%)	Clinical Cases Reporting the Data
Cyst diameter (mean±SD) (mm)	136.3±41.5	43
Cyst growth during pregnancy	22 (52)	42
Surgery during gestation	27 (57)	47
In emergency settings	6/27 (22)	
Cyst rupture	2/6 (33)	
Hemorrhage	4/6 (67)	
Postpartum surgery	20 (43)	47
In emergency settings	3/20 (15)	
Cyst rupture	2/3 (67)	
Hemorrhage	1/3 (33)	
Type of surgery		42
Distal splenopancreatectomy	27 (64)	
Pancreaticoduodenectomy	8 (19)	
Total pancreatectomy	3 (7)	
Enucleation	3 (7)	
Abdominal exploration	1 (2)	
Surgical outcomes		32
Biochemical leak	1 (3)	
POPF grade B	1 (3)	
POPF grade C	0	
Biliary fistula	0	
Postoperative hemorrhage	0	
Pulmonary embolism	1 (3)	
Spleen infarction	1 (3)	
Length of hospital stay (mean±SD) (d)	10.7±5.2	18
Pregnancy outcome		42
Healthy newborn, vaginal delivery	9 (21)	
Healthy newborn, cesarean section	12 (29)	
Healthy newborn, unspecified delivery	15 (36)	
Spontaneous abortion	4 (9)	
Voluntary termination	2 (5)	
Pathology report		47
MCN: low-grade dysplasia	16 (34)	
MCN: high-grade dysplasia	2 (4)	
Mucinous cystadenocarcinoma	11 (23)	
SPN	13 (28)	
SPN with invasive component	2 (4)	
GIST	1 (2)	
Pancreatic hemangioma	1 (2)	
Pancreatic lymphangioma	1 (2)	

* 43 over 47 clinical cases reported the gestational trimester at diagnosis.

GIST indicates gastrointestinal stromal tumor; MCN, mucinous cystic neoplasm; POPF, postoperative pancreatic fistula; SPN, solid pseudopapillary neoplasm.

The outcomes and the pathology reports stratified for gestational trimesters are shown in Table 3. All of the patients (n=9) with a pancreatic cystic diagnosis during the third trimester of pregnancy were treated with a pancreatic resection after delivery, 3^21^,^24^,^33^ of them were treated in emergency settings after a cesarean section. The mean diameter of the cyst was 123.7±43.6, 133.4±42.1, and 152.6±35.2 in first, second, and third trimester, respectively. Emergency surgery was necessary for 3 patients (21%) in the first trimester, 3 (15%) in the second trimester, and 3 (33%) in the third trimester.

**TABLE 3:** Outcomes by Gestational Trimester

	First Trimester (N=14)*	Second Trimester (N=20)*	Third Trimester (N=9)*
Cyst diameter (mean±SD) (mm)	123.7±43.6	133.4±42.1	152.6±35.2
Cyst growth during pregnancy	5 (36)	8 (40)	7 (78)
Surgery during gestation	12 (86)	14 (70)	0
In emergency settings	3/12 (25)	3/14 (21)	0
Cyst rupture	1/3 (33)	1/3 (33)	0
Hemorrhage	2/3 (67)	2/3 (67)	0
Postpartum surgery	2 (14)	6 (30)	9 (100)
In emergency settings	0	0	3/9 (33)
Cyst rupture	0	0	2/3 (67)
Hemorrhage	0	0	1/3 (33)
Type of surgery			
Distal splenopancreatectomy	9 (64)	11 (55)	4 (44)
Pancreaticoduodenectomy	2 (14)	5 (25)	1 (11)
Total pancreatectomy	1 (7)	1 (5)	1 (11)
Enucleation	0	2 (10)	1 (11)
Abdominal exploration: biopsy	0	0	1 (11)
Surgical outcomes			
Biochemical leak	0	0	1 (11)
POPF grade B	0	1 (5)	0
POPF grade C	0	0	0
Biliary fistula	0	0	0
Postoperative hemorrhage	0	0	0
Pulmonary embolism	0	0	1 (11)
Spleen infarction	0	1 (5)	0
Length of hospital stay (mean±SD) (d)	9.8±4.3	11.4±6.2	10.7±4
Pregnancy outcome			
Healthy newborn, vaginal delivery	3 (21)	4 (20)	2 (22)
Healthy newborn, cesarean section	1 (7)	8 (40)	3 (33)
Healthy newborn, unspecified delivery	5 (36)	5 (25)	2 (22)
Abortion	2 (14)	1 (5)	1 (11)
Voluntary termination	1 (7)	1 (5)	0
Pathology report			
MCN: low-grade dysplasia	5 (36)	7 (35)	3 (33)
MCN: high-grade dysplasia	2 (14)	—	—
Mucinous cystadenocarcinoma	3 (21)	1 (5)	4 (44)
SPN	2 (14)	10 (50)	1 (11)
SPN with invasive component	1 (7)	1 (5)	—
GIST	—	1 (5)	—
Hemangioma	1 (7)	—	—
Lymphangioma	—	—	1 (11)

* 43 over 47 clinical cases reported the gestational trimester at diagnosis.

GIST indicates gastrointestinal stromal tumor; MCN, mucinous cystic neoplasm; POPF, postoperative pancreatic fistula; SPN, solid pseudopapillary neoplasm.

## DISCUSSION

PAPC are apparently rare entities, but according to Iacopi et al,^51^ 41% of the 61 pancreatic surgeons responding their survey faced at least 1 PAPC during the course of their career, and 31% of them operated on >1 patient. Therefore, to come across a pregnant woman with a pancreatic cyst is a concrete eventuality for a pancreatic surgeon, especially if working in a high-volume center. Since this systematic review includes only 47 patients, it is plausible that some cases are not reported. As expected, given the study population selection, the majority of the pathology reports described MCN (61%) or SPN (32%) and that 64% of these were located in the body-tail of the gland.

Among the clinical cases reported in the literature, the distal pancreatic resection performed in our institution is the only one carried out by a fully laparoscopic approach. Boyd et al^23^ described a simple laparoscopic abdominal exploration with biopsy because of the advanced stage of the disease. In broad terms, laparoscopic distal resection of the pancreas is a challenging procedure but with recognized advantages over an open operation in terms of length of hospitalization and functional recovery, with comparable morbidity and mortality rates and oncological results.^52^ Regarding abdominal surgery in pregnancy, laparoscopic appendectomy is associated to the higher relative risk of miscarriage compared with open appendectomy,^53^–^55^ whereas laparoscopic cholecystectomy is the gold standard in the treatment of gallstone diseases during pregnancy since it is associated to a lower rate of miscarriage and morbidity compared with the open or conservative approach.^56^ All patients with PAPC diagnosed during the third trimester of pregnancy were operated after delivery, and in this situation, particularly when the tumor is located in the body-tail of the gland, it appears safe to offer a laparoscopic approach. Other settings in which a laparoscopic resection might be safely offered is when pregnancy is voluntarily terminated or spontaneous abortion occurs. Given the lack of data, the technical difficulties due to the size of the cystic tumor and uterus, and concerns for iatrogenic miscarriage suggest caution for laparoscopic pancreatic resection during pregnancy. However, when a cystic neoplasm is diagnosed during the first or second trimester of pregnancy, delaying surgery after delivery is not always an option, and the decision should be based on symptomatology, estimated risk of malignancy, cyst rupture, bleeding, and fetus death.

The mean diameter of the cysts reported in the available literature was considerably large, and in 9% of the cases, a spontaneous cyst rupture was described. Particularly in case of MCN, it is important to avoid tumor rupture because of the potential risk of peritoneal dissemination.^57^

In 52% of the cases, the pancreatic cysts increased in size during pregnancy, 86% of the growing cysts were MCN, and the remaining were SPN. Size is one of the main features associated with the malignant degeneration of MCN. The risk of malignancy should be assessed preoperatively with magnetic resonance imaging and endoscopic ultrasound with fine-needle aspiration of the fluid for cytology and CEA and CA 19-9 assay.^58^ The 2018 European evidence-based guidelines on pancreatic cystic neoplasms^4^ recommend surgical resection for MCN ≥4 cm in diameter. The mean cyst diameter in this review was 136 mm, and one third of the MCN diagnosed during pregnancy were malignant or with high-grade dysplasia. The malignancy rate of resected MCN in nonpregnant subjects is considerably lower (7% to 15%).^59^–^61^ MCN origin is still debated, but these tumors are characterized by an ovarian-type stroma which regularly expresses progesterone receptors, suggesting a hormone involvement in their pathogenesis.^62^ The hypothesis seeing pregnancy involved in MCN growth and eventually in malignant degeneration might be considered.

The major limitation to this literature review is the scarce availability of studies addressing PAPC. All of the studies included in the review are clinical cases or case series. Consequently, the cohort of patients analyzed in the study has the chance of suffering a selection bias. Because of the low quality of the studies available in the literature, no strong recommendation may be posed. This limit might be partially overcome by the creation of a multicenter pancreatic cystic neoplasm registry to better understand the possible interactions between pancreatic cystic neoplasms and pregnancy.

In conclusion, the most worrisome complications of PAPC requiring emergency surgical treatment are bleeding and cyst rupture. MCN are the most common PAPC, they have a tendency to grow during pregnancy, and an increased risk of malignant degeneration might be considered. When the diagnosis occurred during the third trimester of pregnancy, surgery was always postponed after delivery. This study is the first to report a laparoscopic pancreatic resection in a patient bearing a PAPC.

## Supplementary Material

Supplemental Digital Content is available for this article. Direct URL citations appear in the printed text and are provided in the HTML and PDF versions of this article on the journal's website, www.surgical-laparoscopy.com.
