# Supplementary material for: Pancreatic Cystic Neoplasms and Pregnancy: A Systematic Review of Surgical Cases and a Case Report of a Fully Laparoscopic Distal Pancreatectomy
Source: Surg Laparosc Endosc Percutan Tech. 2021 Dec 9;32(1):133–9. doi: 10.1097/SLE.0000000000001023 (PMC9907686; doi:10.1097/SLE.0000000000001023)
Supplement: Supplementary file 1 [file sle-32-133-s001.docx]

**Supplementary table 1 – Summary of pregnancy associated pancreatic cystic neoplasms reported in literature**

| Authors | Year | Country | Age | Gestational trimester | Cyst size (mm) | Complication | Histology | Pregnancy outcome |
| --- | --- | --- | --- | --- | --- | --- | --- | --- |
| Fogliati A et al. (current case) | 2021 | Italy | 25 | Third | 180 | - | Low grade MCN | Successful delivery |
| Santos D et al.^6^ | 2020 | Portugal | 23 | Second | 140 | - | Invasive SPN | Successful delivery |
| Revoredo F et al.^7^ | 2020 | Peru | 38 | Second | 200 | Cyst rupture | Low grade MCN | Successful delivery |
| Revoredo F et al.^7^ | 2020 | Peru | 30 | Second | 118 | - | Mucinous cystadenocarcinoma | Successful delivery |
| Carvahlo L et al.^8^ | 2020 | Portugal | 32 | Third | 210 | - | Low grade MCN | Successful delivery |
| Tanacan A et al.^9^ | 2018 | Turkey | 26 | Second | 90 | - | SPN | Successful delivery |
| Huang TT et al.^10^ | 2018 | China | 26 | Second | 124 | - | SPN | Successful delivery |
| Santamaria-Barria JA et al.^11^ | 2017 | USA | 20 | Second | 150 | - | GIST | Successful delivery |
| Al-Umair RS el al.^49^ | 2015 | Oman | 34 | Third | 137 | - | SPN | Successful delivery |
| Soreide JA et al.^12^ | 2015 | Norway | 30 | First | 170 | - | Hemangioma | Successful delivery |
| Kosumi K et al.^13^ | 2015 | Japan | 33 | First | 76 | - | Low grade MCN | Successful delivery |
| Yee AM et al.^14^ | 2015 | USA | 39 | Second | 112 | - | SPN | Successful delivery |
| Sharanappa V et al.^16^ | 2015 | India | 22 | Second | 120 | - | SPN | Voluntary termination |
| Kleeff J et al.^17^ | 2015 | Germany | 41 | Second | 70 | - | Low grade MCN | - |
| Macdonald F et al.^18^ | 2014 | Canada | 23 | Second | 150 | - | SPN | Successful delivery |
| Tica AA et al.^15^ | 2013 | Romania | 27 | Third | 149 | - | Low grade MCN | Successful delivery |
| Huang SC et al.^19^ | 2013 | Taiwan | 29 | Second | - | Hemorrhage | SPN | Successful delivery |
| Tsuda H et al.^20^ | 2012 | Japan | 28 | First | 140 | - | High grade MCN | Successful delivery |
| Boumans D et al.^21^ | 2012 | Netherlands | 30 | Third | 185 | Hemorrhage | Low grade MCN0 | Abortion |
| Iusco DR et al.^22^ | 2012 | Italy | 28 | - | 160 | - | Mucinous cystadenocarcinoma | Successful delivery |
| Boyd CA et al.^23^ | 2011 | USA | 21 | First | 172 | - | Low grade MCN | Successful delivery |
| Boyd CA et al.^23^ | 2011 | USA | 29 | Third | - | - | Mucinous cystadenocarcinoma | Successful delivery |
| Naganuma S et al.^24^ | 2011 | Japan | 32 | Third | 110 | Cyst rupture | Mucinous cystadenocarcinoma | Successful delivery |
| Feng JF et al.^25^ | 2011 | China | 26 | Second | 95 | - | SPN | Successful delivery |
| Asciutti S et al.^26^ | 2010 | Italy | 31 | Second | 85 | - | Low grade MCN | Successful delivery |
| Brown TH et al.^27^ | 2009 | UK | 38 | First | 100 | Hemorrhage | High grade MCN | Successful delivery |
| Hajdu N et al.^28^ | 2009 | Hungary | 29 | Second | 160 | - | SPN | Successful delivery |
| Ikuta SI et al.^29^ | 2008 | Japan | 30 | First | 180 | - | Low grade MCN | Abortion |
| Hakamada K et al.^30^ | 2008 | Japan | 38 | First | 100 | Hemorrhage | Mucinous cystadenocarcinoma | Successful delivery |
| Wiseman JES et al.^31^ | 2008 | USA | 32 | Second | 167 | - | Low grade MCN | Successful delivery |
| Berindoague R et al.^32^ | 2007 | Spain | 31 | - | 120 | - | Mucinous cystadenocarcinoma | Successful delivery |
| Ozden S et al.^33^ | 2007 | Turkey | 32 | Third | 150 | Cyst rupture | Mucinous cystadenocarcinoma | Successful delivery |
| Herring AA et al.^34^ | 2007 | USA | 34 | First | 190 | - | Mucinous cystadenocarcinoma | Successful delivery |
| Ishikawa K et al.^35^ | 2007 | Japan | 33 | Second | 180 | - | Low grade MCN | Successful delivery |
| Kitagawa H et al.^36^ | 2006 | Japan | 25 | - | 150 | - | Low grade MCN | Successful delivery |
| Kato M et al.^39^ | 2005 | Japan | 33 | Second | 220 | - | Low grade MCN | Successful delivery |
| Lopez-Tomassetti Fernandez EM et al.^40^ | 2005 | Spain | 26 | Second | 140 | - | Low grade MCN | Successful delivery |
| Matsunaga T et al.^37^ | 2005 | Japan | 28 | - | 200 | - | Mucinous cystadenocarcinoma | - |
| Levy C et al.^38^ | 2004 | USA | - | Second | 80 | - | SPN | Successful delivery |
| Ganepola GA et al.^41^ | 1999 | USA | 37 | First | 120 | - | Low grade MCN | - |
| Morales A et al.^42^ | 1998 | Mexico | 21 | First | 120 | - | Invasive SPN | Successful delivery |
| Olsen ME et al.^43^ | 1993 | USA | 25 | First | 50 | - | Low grade MCN | Successful delivery |
| Orlando CA et al.^44^ | 1991 | USA | - | First | 90 | - | SPN | - |
| Baiocchi C et al^45^ | 1990 | Italy | 29 | Third | 100 | - | Mucinous cystadenocarcinoma | Successful delivery |
| Bondeson AG et al.^46^ | 1990 | USA | 19 | First | - | - | SPN | Abortion |
| Smithers BM et al.^48^ | 1986 | UK | 33 | First | 100 | Cyst rupture | Mucinous cystadenocarcinoma | Voluntary termination |
| Duff P et al.^47^ | 1985 | USA | 35 | Second | - | Hemorrhage | SPN | Abortion |

MCN = mucinous cystic neoplasm, SPN = solid pseudopapillary neoplasm, GIST = gastrointestinal stromal tumor.
